# The association between malnutrition status and hemorrhagic transformation in patients with acute ischemic stroke receiving intravenous thrombolysis

**DOI:** 10.1186/s12883-023-03152-3

**Published:** 2023-03-14

**Authors:** Yerim Kim, Minwoo Lee, Hee Jung Mo, Chulho Kim, Jong-Hee Sohn, Kyung-Ho Yu, Sang-Hwa Lee

**Affiliations:** 1 Department of Neurology, Kangdong Sacred Heart Hospital, Hallym University College of Medicine, Seoul, South Korea; 2 Department of Neurology, Hallym Sacred Heart Hospital, Hallym University College of Medicine, Anyang, South Korea; 3 Department of Neurology, Dongtan Sacred Heart Hospital, Hallym University College of Medicine, Hwaseong, South Korea; 4 Department of Neurology, Chuncheon Sacred Heart Hospital, Hallym University College of Medicine, 77 Sakju-ro, Chuncheon, South Korea; 5 Institute of New Frontier Research Team, Hallym University, Chuncheon, South Korea

**Keywords:** Malnutrition, CONUT score, PNI, Intravenous thrombolysis, Hemorrhagic transformation

## Abstract

**Objectives:**

We evaluated the impact of malnutrition as estimated by the controlling nutritional status (CONUT) score and prognostic nutritional index (PNI) on hemorrhagic transformation (HT) and stroke outcomes after intravenous thrombolysis (IVT).

**Materials and methods:**

Using a multicenter registry database, we enrolled 808 patients with acute ischemic stroke who received IVT between August 2013 and May 2021. We defined malnutrition as a CONUT score ≥ 2 and low PNI. The primary outcome measure was the occurrence of symptomatic HT contributing to early neurologic deterioration (END-SHT) after IVT. Multivariable analysis was performed to analyze the association between CONUT score, PNI, and END-SHT after IVT.

**Results:**

The rate of END-SHT was higher with increasing CONUT scores and PNI values. In the multivariable analysis, CONUT score ≥ 5 and low PNI were significantly associated with END-SHT (odds ratio [95% confidence interval], CONUT score ≥ 5: 12.23 [2.41–62.07], p = 0.003; low PNI: 4.98 [1.76–14.09], p = 0.003). The receiver operating characteristic curve showed that both the CONUT score and PNI had good predictive ability. The cutoff values for CONUT and PNI were 5 and 42.3, respectively, for END-SHT.

**Conclusion:**

Malnutrition, as denoted by a higher CONUT score and lower PNI, was associated with END-SHT. The joint application of both nutritional markers could be useful in predicting END-SHT after IVT.

**Supplementary Information:**

The online version contains supplementary material available at 10.1186/s12883-023-03152-3.

## Introduction

The prevalence of malnutrition in acute stroke has been estimated to range from 12.2–62% [1, 2]. It is generally accepted that malnutrition affecting ischemic stroke patients have been negatively associated with several clinical outcomes [3–9]. However, the prevalence of malnutrition after stroke varies widely, due to heterogenous study subjects, stroke subtypes, varying nutritional parameters, and differing definitions of malnutrition. Nonetheless, since early nutritional assessment could improve clinical outcomes in acute ischemic stroke,[10] its importance particularly increases during the early stages of stroke.

Nutritional interventions for acute ischemic stroke have received little attention in real-world practice. The difficulties in assessing nutritional status in stroke patients could explain this situation. Weight and dietary history may not be captured from patients and their kin; simple assessments such as body mass index (BMI) were not always available in immobile stroke patients [2]. Notably, objective nutritional markers, such as the controlling nutritional status (CONUT) score and the prognostic nutritional index (PNI), were easy to calculate using serum albumin, cholesterol levels, and lymphocyte count in peripheral blood and could be feasible in clinical practice [11].

Given that the CONUT score and PNI represent nutrition and immune response, we assumed that the scores at hospitalization could predict the outcomes of stroke patients receiving intravenous thrombolysis (IVT). Since the CONUT score and PNI at hospitalization were less likely to be affected by neurological disability, the score could represent pre-thrombolysis nutritional status [12, 13]. However, only a few studies have evaluated the impact of nutritional status on stroke outcomes after IVT [13]. Using a multicenter database, we aimed to investigate the impact of the CONUT score and PNI on hemorrhagic transformation (HT) and stroke outcomes in patients receiving IVT.

## Materials and methods

### Subjects

We consecutively registered all patients with acute ischemic stroke between August 2013 and May 2021 at four university-affiliated institutions (Hallym Sacred Heart Hospital, Chuncheon Sacred Heart Hospital, Kangdong Sacred Heart Hospital, and Dongtan Sacred Heart Hospital). In this study, we identified patients with acute ischemic stroke who were treated with IVT. All included patients received intravenous recombinant tissue plasminogen activator (tPA) according to the current guidelines for acute stroke management. According to the imaging protocols of each institution, we routinely performed follow-up brain computed tomography (CT) or magnetic resonance imaging (MRI) within 24 h after IVT. Additionally, these imaging tools were used when patients experienced neurological deterioration. We excluded the following patients: (1) patients with unavailable CONUT score and PNI; (2) patients without follow-up brain CT or MRI within 24 h of stroke onset; (3) patients who were not available for assessment of early neurological deterioration (END), HT after IVT, and the modified Rankin Scale (mRS) at 3 months; (4) patients with a pre-stroke mRS score ≥ 2; (5) patients who had additional endovascular interventional therapy after IVT and (6) patients who had active malignancy, infection/inflammation, hematologic disease, or infusion of blood products within 24 h after IVT.

### Clinical Data collection and definition of parameters

The following data were directly obtained from the registry database: (1) demographics, including age and sex; (2) medical history, including stroke risk factors, prior stroke, hypertension, diabetes mellitus (DM), coronary artery disease, hyperlipidemia, atrial fibrillation, current smoking status, pre-stroke status, and prior use of statins and antithrombotic drugs; (3) stroke characteristics, acute stroke treatment, initial NIHSS score, and ischemic stroke mechanism according to the Trial of Org 10,172 in Acute Stroke Treatment (TOAST) classification with some modifications,[14] tPA dose, and reperfusion therapy (IVT and intra-arterial thrombectomy [IAT]); and (4) laboratory data sampled within 12 h of stroke onset (median time of interval from stroke onset to sample acquisition 2.9 h [interquartile range 1.3-6.0]).

### Malnutrition tools

The CONUT score was estimated using the serum albumin level, lymphocyte count, and total cholesterol level. The detailed parameters and scoring system for the CONUT score are described in Supplementary Table 1. According to the traditional stratification of the CONUT scoring system, a CONUT score of 2–4 was mild malnutrition, 5–8 was moderate malnutrition, and 9–12 was severe malnutrition [11]. The PNI score was estimated using the formula: 5x lymphocyte count (10^9^/L) + 10 × serum albumin (g/dL).

### Outcome measures

END was defined as an increment of at least 1 point in motor power or a total NIHSS score deterioration of ≥ 2 points within 7 days of admission compared to the initial NIHSS score [15]. We categorized the etiology of END as follows: (1) symptomatic hemorrhagic transformation (END-SHT) and (2) stroke progression (END-prog) after IVT [16]. With respect to the definition of END, the primary outcome measures were the occurrence of symptomatic hemorrhagic transformation contributing to early neurologic deterioration (END-SHT) and any HT after IVT. END-SHT was defined as the presence of HT, which was defined using the European Cooperative Acute Stroke Study criteria based on follow-up brain CT or MR images [17]. Any HT was defined as a new bleeding lesion on follow-up images after IVT. Secondary outcome measures were stroke progression (END-prog) and good functional outcome at 3 months (mRS 0 to 2) [18]. END-prog was defined as END caused by the progression of the initial ischemic lesion, which occurred due to the enlargement of infarct size or the presence of significant perilesional edema on follow-up images [15]. Two vascular neurologists (M Lee and S-H Lee) reviewed the END data to confirm END-SHT and END-prog in a double-blinded manner (interclass correlation coefficient, 0.88; p < 0.001 in END-SHT and 0.89; p < 0.001 in END-prog).

### Statistical analysis

We hypothesized that malnutrition (high CONUT score and low PNI) would increase the risk of END-SHT and poor functional outcomes after IVT. With respect to the primary and secondary outcome measures, the categorized CONUT score (normal, mild, and moderate to severe) and PNI were compared using Pearson’s chi-squared test for categorical variables and Student’s t-test or Mann-Whitney U test for continuous variables. We dichotomized PNI as low and high groups based on a cut-off value calculated by receiver operating characteristic (ROC) curve analysis. We performed a binary logistic regression analysis to evaluate the independent effects of the categorized CONUT score on stroke outcomes using clinically plausible variables or univariate p < 0.10. Crude and adjusted odds ratios (ORs) and 95% confidence intervals (CIs) were calculated.

We performed a ROC curve to determine the predictive ability and optimal cutoff value of the CONUT score and PNI on END-SHT, using the ‘pROC’ package of R. the Youden index estimated the cut-off values of the CONUT score and PNI for END-SHT. Statistical analyses were performed using IBM SPSS version 21.0 software (IBM Corporation, Armonk, NY, USA) and R version 4.0.3 (R core Team 2020; R Foundation for Statistical Computing, Vienna, Austria).

## Results

Among 10,808 consecutive patients with acute ischemic stroke, 1,259 underwent IVT and IAT. Of the 1,259 patients, 808 who received IVT alone were included in our study. The mean age of these patients was 66.7 (± 13.3) years, 513 (63.5%) were male, the median CONUT score was 1 (0–2), and the median PNI was 52.5 (47.1–57.5). With the respect to the CONUT scoring system, the proportion of malnutrition in acute ischemic stroke patients was 39.6% (mild, 34.2%; moderate to severe, 5.4%). We established a PNI value higher than 42.3 as the optimal cut-off point for END-SHT. Based on this criteria, patients were divided into low and high PNI groups. According to the CONUT scoring system, patients with moderate-to-severe malnutrition were likely to be older and had higher stroke severity, previous stroke, and DM history. Interestingly, the groups with moderate-to-severe malnutrition, whether through the CONUT or PNI scoring system, had more large artery atherosclerosis (LAA) and cardioembolism stroke subtypes and fewer small vessel occlusion (SVO) stroke subtypes in our study. The baseline demographic and clinical characteristics are described in Tables 1 and 2, respectively.

**Table 1:** Baseline characteristics according to CONUT scoring system

	Normal (CONUT 0–1) (n = 488)	Mild (CONUT 2–4) (n = 276)	Moderate to severe (CONUT 5–12) (n = 44)	p-value
Age (SD)	65.2 (12.7)	68.6 (13.8)	71.3 (13.8)	< 0.001
Male, n (%)	311 (63.7)	178 (64.5)	24 (54.5)	0.44
NIHSS (IQR)	7 (5–11)	8 (5–14)	8 (6–13)	0.02
BMI, kg/m^2^ (SD)	24.0 (22.0-25.7)	23.7 (21.2–25.6)	22.3 (20.8–24.6)	0.01
Stroke mechanism, n (%)				0.04
SVO	89 (18.2)	29 (10.5)	3 (6.8)	
LAA	146 (29.9)	81 (29.3)	14 (31.8)	
CE	132 (27.0)	95 (34.4)	126 (36.4)	
UD	106 (21.7)	63 (22.8)	11 (25.0)	
OD	15 (3.1)	8 (2.9)	0 (0.0)	
Interval from arrival to IVT, min (IQR)	39 (28–54)	40 (31–55)	44 (36-55.5)	0.22
Previous stroke, n (%)	73 (15.0)	52 (18.8)	14 (31.8)	0.01
HTN, n (%)	292 (59.8)	163 (59.1)	25 (56.8)	0.92
DM, n (%)	112 (23.0)	101 (36.6)	15 (34.1)	< 0.001
HL, n (%)	92 (18.9)	53 (19.2)	10 (22.7)	0.83
CAD, n (%)	28 (5.3)	44 (15.9)	5 (11.4)	< 0.001
Current smoking, n (%)	113 (23.2)	52 (18.8)	8 (18.2)	0.33
Atrial fibrillation, n (%)	132 (27.0)	94 (34.1)	18 (40.9)	0.04
Previous use of antithrombotics, n (%)	116 (23.8)	93 (33.7)	11 (25.0)	0.01
tPA dose, n (%)				0.002
0.6 mg/kg	73 (15.0)	17 (6.2)	4 (9.1)	
0.9 mg/kg	415 (85.0)	259 (93.8)	40 (90.9)	
Total cholesterol, mg/dL (SD)	190.8 (35.5)	134.9 (28.5)	112.3 (14.4)	< 0.001
Albumin, mg/dL (SD)	4.2 (0.4)	4.1 (0.4)	2.8 (0.8)	< 0.001
Hemoglobin, g/dL (SD)	13.9 (2.0)	13.5 (1.9)	13.2 (2.1)	0.001
Creatinine, mg/dL (SD)	0.93 (0.32)	1.11 (0.79)	1.10 (0.49)	< 0.001
Platelet, x1000/µL (SD)	235.7 (68.9)	215.9 (70.6)	221.1 (77.0)	0.001
LDL, mg/dL (SD)	118.1 (30.2)	85.4 (29.8)	97.8 (41.8)	< 0.001
HbA1c, % (SD)	6.1(1.2)	6.1 (1.0)	6.0 (1.2)	0.37
Prothrombin time, INR (SD)	1.01 (0.11)	1.04 (0.14)	1.04 (0.17)	0.01
CRP, mg/dL (SD)	6.8 (14.1)	8.3 (15.6)	12.8 (39.7)	0.054
Initial random glucose, mg/dL (SD)	142.4 (53.0)	147.9 (64.1)	136.6 (61.5)	0.30
SBP, mmHg (SD)	155.1 (28.1)	148.6 (25.5)	156.4 (25.5)	0.01
PNI, (IQR)	54.6 (50.6–59.5)	48.8 (42.2–54.0)	31.6 (29.9–37.4)	< 0.001

Abbreviation: CONUT, controlling nutritional status; SD, standard deviation; NIHSS, National Institute Health of Stroke Scale; BMI, body mass index; IQR, interquartile range; SVO, small vessel occlusion; LAA, large artery atherosclerosis; CE, cardioembolism; UD, undetermined; OD, other determined; IVT, intravenous thrombolysis; HTN, hypertension; DM, diabetes mellitus; HL, hyperlipidemia; CAD, coronary artery disease; tPA, tissue plasminogen activator; LDL, low density lipoprotein; HbA1c, glycated hemoglobin; INR, international normalized ratio; CRP, C reactive protein; SBP, systolic blood pressure; PNI, prognostic nutritional index

**Table 2:** Baseline characteristics according to PNI.

	low PNI ≤ 42.3 (n = 116)	high PNI > 42.3 (n = 692)	p-value
Male, n (%)	68 (58.6)	445 (64.3)	0.25
NIHSS (IQR)	10 (6–16)	7 (5–12)	< 0.001
BMI, kg/m^2^ (SD)	23.4 (21.4–24.8)	23.9 (21.8–25.7)	0.04
SVO	6 (5.2)	115 (16.6)	
LAA	41 (35.3)	200 (28.9)	
CE	38 (32.8)	205 (29.6)	
UD	28 (24.1)	152 (22.0)	
OD	3 (2.8)	20 (2.9)	
Interval from arrival to IVT, min (IQR)	42 (35–56)	39 (29–54)	0.09
Previous stroke, n (%)	23 (19.8)	116 (16.8)	0.43
HTN, n (%)	68 (58.6)	412 (59.5)	0.92
CAD, n (%)	14 (12.1)	61 (8.8)	0.30
Current smoking, n (%)	22 (19.0)	151 (21.8)	0.54
Atrial fibrillation, n (%)	44 (37.9)	200 (28.9)	0.06
Previous use of antithrombotics, n (%)	25 (21.6)	195 (28.2)	0.15
tPA dose, n (%)			0.06
0.6 mg/kg	7 (6.0)	87 (12.6)	
0.9 mg/kg	109 (94.0)	605 (87.4)	
Total cholesterol, mg/dL (SD)	151.0 (44.9)	173.1 (42.3)	0.21
Albumin, mg/dL (SD)	3.0 (0.7)	4.2 (0.4)	< 0.001
Hemoglobin, g/dL (SD)	13.5 (2.0)	13.8 (2.0)	0.16
Creatinine, mg/dL (SD)	1.16 (0.88)	0.97 (0.46)	0.002
Platelet, x1000/µL (SD)	222.3 (73.1)	229.1 (70.1)	0.32
LDL, mg/dL (SD)	103.5 (38.4)	106.2 (33.7)	0.03
HbA1c, % (SD)	6.0 (2.0)	6.1 (1.1)	0.27
Prothrombin time, INR (SD)	1.04 (0.16)	1.02 (0.12)	0.35
CRP, mg/dL (SD)	11.3 (27.8)	7.0 (14.4)	0.001
Initial random glucose, mg/dL (SD)	151.5 (80.9)	142.7 (52.6)	0.004
SBP, mmHg (SD)	153.3 (24.7)	152.9 (27.7)	0.16

Abbreviation: PNI, prognostic nutritional index; SD, standard deviation; NIHSS, National Institute Health of Stroke Scale; BMI, body mass index; IQR, interquartile range; SVO, small vessel occlusion; LAA, large artery atherosclerosis; CE, cardioembolism; UD, undetermined; OD, other determined; IVT, intravenous thrombolysis; HTN, hypertension; DM, diabetes mellitus; HL, hyperlipidemia; CAD, coronary artery disease; tPA, tissue plasminogen activator; LDL, low density lipoprotein; HbA1c, glycated hemoglobin; INR, international normalized ratio; CRP, C reactive protein; SBP, systolic blood pressure;

**Fig. 1:**
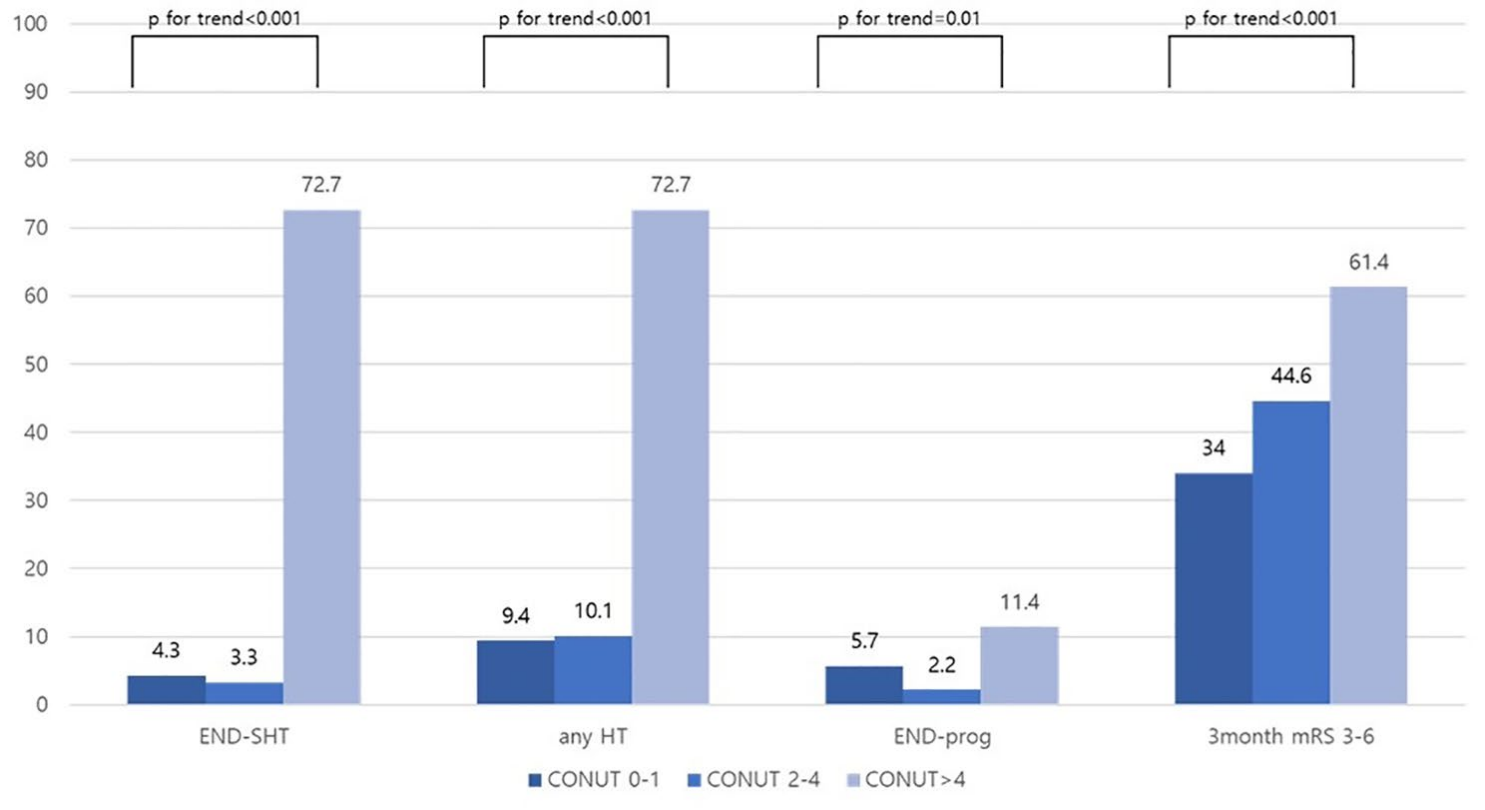
The distribution of stroke outcomes according to CONUS score Abbreviations: CONUT, controlling nutritional status; END-SHT, symptomatic hemorrhagic transformation; HT, hemorrhagic transformation; END-prog, stroke progression; mRS, modified Rankin Scale.

**Fig. 2:**
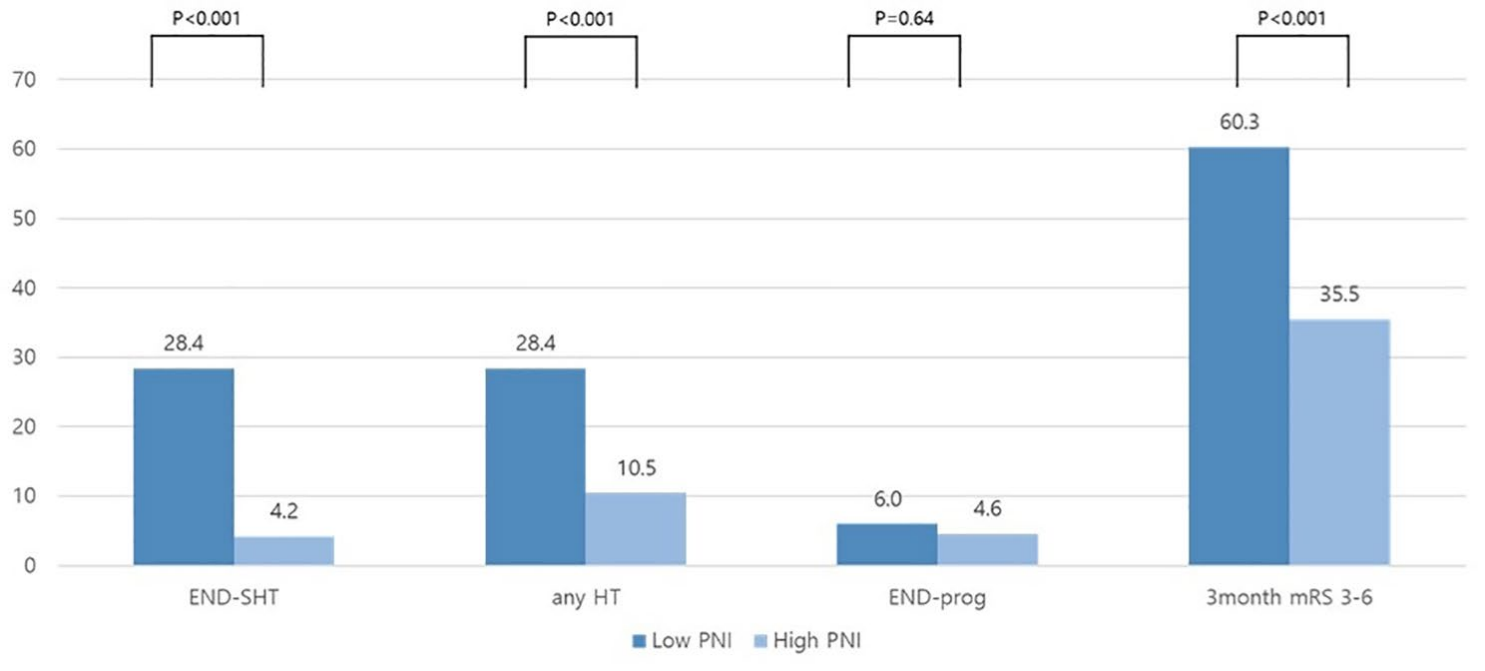
The distribution of stroke outcomes according to PNI score Abbreviations: PNI, prognostic nutritional index; END-SHT, symptomatic hemorrhagic transformation; HT, hemorrhagic transformation; END-prog, stroke progression; mRS, modified Rankin Scale.

For the main outcome, the rates of END-SHT and HT were higher with increasing CONUT scores (END-SHT: normal; 21 (4.3%), mild; 9 (3.3%) and moderate to severe; 32 (72.7%), any HT: normal; 46 (9.4%), mild; 28 (10.1%) and moderate to severe; 32 (72.7%), p for trend < 0.001 Fig. 1). The low PNI group also had higher rates of END-SHT and HT than the high PNI group (END-SHT: 33 (28.4%) versus 29 (4.2%); any HT: 33 (28.4%) versus 73 (10.5%); p < 0.001; Fig. 2). For the secondary outcomes, the rates of END-prog [normal; 28 (5.7%), mild; 6 (2.2%) and moderate to severe; 5 (11.4%)] and poor functional outcome at 3 months also increased with higher CONUT scores [normal; 166 (34.0%), mild; 123 (44.6%) and moderate to severe; 27 (61.4%)] (Fig. 1). Similarly, the low PNI group had a higher rate of poor functional outcomes at 3 months than the high PNI group [70 (60.3%) versus 246 (35.5%), p < 0.001, Fig. 2].

In multivariable analysis, moderate to severe nutritional status based on the CONUT scoring system was significantly associated with END-SHT and HT (OR [95% CI], END-SHT: 14.52 [3.15–66.95], p = 0.001; HT: 9.40 [2.74–32.20], p < 0.001). A low PNI was also significantly associated with END-SHT and HT (OR [95% CI], END-SHT: 4.90 [1.72–13.92], p = 0.003; HT: 3.40 [1.46–7.89], p = 0.004). For secondary outcomes, moderate-to-severe malnutrition and low PNI increased the risk of END-prog and poor functional outcomes at 3 months (Tables 3 and 4). In sensitivity analysis, both CONUT and PNI were associated with 3-month mortality (Supplementary Tables 2 and 3).

**Table 3:** Multivariate analysis showing association between CONUT scoring system and outcomes

	END-SHT			Any HT			END-prog			3-month mRS 3–6		
	OR	95% CI	p-value	OR	95% CI	p-value	OR	95% CI	p-value	OR	95% CI	p-value
CONUT scoring system												
normal	ref			ref			ref			ref		
mild	0.57	0.20–1.62	0.30	0.85	0.43–1.68	0.64	0.34	0.12–1.03	0.057	1.27	0.78–2.05	0.34
moderate to severe	12.23	2.41–62.07	0.003	7.90	2.20–28.40	0.002	3.01	1.51–17.79	0.03	3.10	1.12–8.55	0.03
Age	1.02	0.98–1.05	0.34	1.01	0.99–1.03	0.43	1.03	0.99–1.06	0.19	1.05	1.03–1.07	< 0.001
Male	0.87	0.39–1.98	0.75	1.22	0.69–2.17	0.49	1.28	0.57–2.84	0.55	0.77	0.51–1.12	0.16
NIHSS	1.02	0.95–1.09	0.66	1.01	0.97–1.06	0.57	1.05	0.99–1.12	0.08	1.16	1.12–1.20	< 0.001
Stroke mechanism												
SVO	ref			ref			ref			ref		
LAA	0.98	0.26–3.64	0.98	1.17	0.46–2.99	0.74	1.74	0.53–5.79	0.36	1.67	0.94–2.99	0.08
CE	0.70	0.14–3.57	0.67	1.83	0.58–5.75	0.3	1.19	0.18–8.14	0.86	0.95	0.40–2.23	0.91
UD	0.98	0.25–3.89	0.97	1.55	0.60–4.02	0.37	0.35	0.07–1.87	0.22	1.13	0.60–2.13	0.70
OD	1.27	0.11–14.26	0.85	0.79	0.09–7.05	0.83	1.12	0.14–16.24	0.68	1.84	0.62–5.45	0.27
Previous stroke	0.68	0.24–1.96	0.48	0.98	0.50–1.91	0.94	0.37	0.11–1.24	0.11	1.45	0.89–2.38	0.14
DM	0.41	0.16–1.05	0.06	0.79	0.45–1.39	0.41	2.43	1.17–5.03	0.02	1.67	1313 − 2.48	0.01
CAD	1.79	0.54–5.91	0.34	1.47	0.60–3.57	0.40	0.67	0.13–3.51	0.63	1.6	0.83–3.06	0.16
Atrial fibrillation	2.04	0.57–7.28	0.27	1.01	0.54–1.87	0.98	1.55	0.31–7.71	0.60	1.53	0.75–3.11	0.24
Previous use of antithrombotics	1.22	0.49–3.06	0.67	1.01	0.54–1.87	0.98	0.62	0.24–1.58	0.31	0.46	0.29–0.74	0.001
tPA dose,	1.36	0.37–4.98	0.65	3.30	1.06–10.26	0.04	2.10	0.36–5.75	0.48-	0.95	0.55–1.64	0.84
Total cholesterol	0.98	0.96–0.996	0.01	0.98	0.97–0.99	< 0.001	1.00	0.99–1.02	0.70	1.00	0.99–1.01	0.91
Albumin	0.46	0.19–1.11	0.08	0.90	0.49–1.65	0.73	1.57	0.66–3.73	0.31	1.12	0.73–1.73	0.6
Hemoglobin	1.23	0.98–1.55	0.08	1.18	1.01–1.39	0.04	0.82	0.66–1.02	0.07	1.04	0.94–1.16	0.44
Platelet	1.00	0.995–1.01	0.92	1.00	0.996-1.00	0.80	1.00	0.995–1.01	0.98	1.00	0.998-1.00	0.86
Creatinine	1.37	0.73–2.58	0.33	1.04	0.65–1.66	0.89	0.73	0.30–1.80	0.49	1.61	1.12–2.31	0.01
LDL	1.02	1.01–1.04	0.01	1.02	1.01–1.04	< 0.001	1.00	0.98–1.02	0.97	1.01	0.998–1.02	0.14
PT	3.54	0.49–25.12	0.21	0.98	0016-6.06	0.98	1.22	0.10-15.32	0.88	0.75	0.19–2.87	0.67
CRP	1.01	0.996–1.03	0.15	1.01	1.00-1.03	0.045	1.00	0.99–1.02	0.66	1.01	0.998–1.02	0.11

Abbreviation: CONUT, controlling nutritional status; END-SHT, symptomatic hemorrhagic transformation; HT, hemorrhagic transformation; END-prog; stroke progression; mRS, modified Rankin Scale; OR, odds ratio; CI, confidence interval; NIHSS, National Institute Health of Stroke Scale; SVO, small vessel occlusion; LAA, large artery atherosclerosis; CE, cardioembolism; UD, undetermined; OD, other determined; DM, diabetes mellitus; HL, hyperlipidemia; CAD, coronary artery disease; tPA, tissue plasminogen activator; LDL, low density lipoprotein; PT, prothrombin time; CRP, C-reactive protein

**Table 4:** Multivariate analysis showing association between PNI system and outcomes

	END-SHT			Any HT			END-prog			3-month mRS 3–6		
	OR	95% CI	p-value	OR	95% CI	p-value	OR	95% CI	p-value	OR	95% CI	p-value
Low PNI	4.98	1.76–14.09	0.003	3.48	1.50–9.09	0.004	3.52	1.02–12.19	0.047	2.95	1.35–6.46	0.01
Age	0.995	0.97–1.03	0.92	0.999	0.98–1.02	0.96	1.03	0.99–1.06	0.11	1.05	1.03–1.06	< 0.001
Male	1.14	0.57–2.29	0.72	1.48	0.89–2.45	0.13	0.85	0.41–1.75	0.65	0.79	0.55–1.13	0.19
NIHSS	0.98	0.92–1.04	0.43	0.99	0.95–1.03	0.64	1.04	0.98–1.10	0.17	1.14	1.10–1.09	< 0.001
Stroke mechanism												
SVO	ref			ref			ref			ref		
LAA	0.97	0.31–3.02	0.96	1.31	0.58–3.10	0.53	1.44	0.44–4.74	0.55	1.62	0.92–2.88	0.1
CE	0.68	0.15–3.04	0.62	1.89	0.64–5.60	0.25	0.74	0.12–4.43	0.74	0.89	0.39–2.06	0.79
UD	1.16	0.36–3.72	0.80	1.73	0.72–4.15	0.22	0.32	0.07–1.58	0.16	1.18	0.64–2.17	0.6
OD	0.39	0.04–4.17	0.44	0.36	0.04–3.36	0.37	0.47	0.11–5.21	0.47	1.54	0.52–4.60	0.44
Interval from arrival to IVT	1.01	0.998–1.01	0.16	1.001	0.995–1.01	0.70	1.00	0.995–1.01	0.39	1.00	0.997–1.01	0.52
Atrial fibrillation	2.19	0.68–7.02	0.19	1.52	0.65–3.55	0.34	1.83	0.43–7.87	0.42	1.33	0.68–2.63	0.41
tPA dose	1.34	0.42–4.34	0.62	2.7	1.02–7.16	0.046	1.12	0.87–8.26	0.51	0.93	0.54–1.58	0.78
Albumin	0.23	0.11–0.51	< 0.001	0.45	0.25–0.78	0.01	1.58	0.72–3.46	0.26	1.21	0.79–1.84	0.39
Creatinine	0.99	0.60–1.64	0.97	0.88	0.59–1.33	0.56	0.71	0.30–1.65	0.42	1.49	1.05–2.12	0.03
LDL	1.01	0.996–1.01	0.31	1.00	0.996–1.01	0.43	1.01	0.995–1.02	0.32	1.00	0.999–1.01	0.15
CRP	1.01	0.99–1.02	0.29	1.01	0.997–1.02	0.15	1.00	0.99–1.02	0.64	1.01	0.996–1.02	0.28
IRG	1.00	0.996–1.01	0.68	1.00	0.998–1.01	0.31	1.00	0.99–1.01	0.73	1.00	1.00-1.01	0.10

Abbreviation: PNI, prognostic nutritional index; END-SHT, symptomatic hemorrhagic transformation; HT, hemorrhagic transformation; END-prog; stroke progression; mRS, modified Rankin Scale; OR, odds ratio; CI, confidence interval; NIHSS, National Institute Health of Stroke Scale; SVO, small vessel occlusion; LAA, large artery atherosclerosis; CE, cardioembolism; UD, undetermined; OD, other determined; IVT, intravenous thrombolysis; tPA, tissue plasminogen activator; LDL, low density lipoprotein; CRP, C-reactive protein; IRG, initial random glucose

**Fig. 3:**
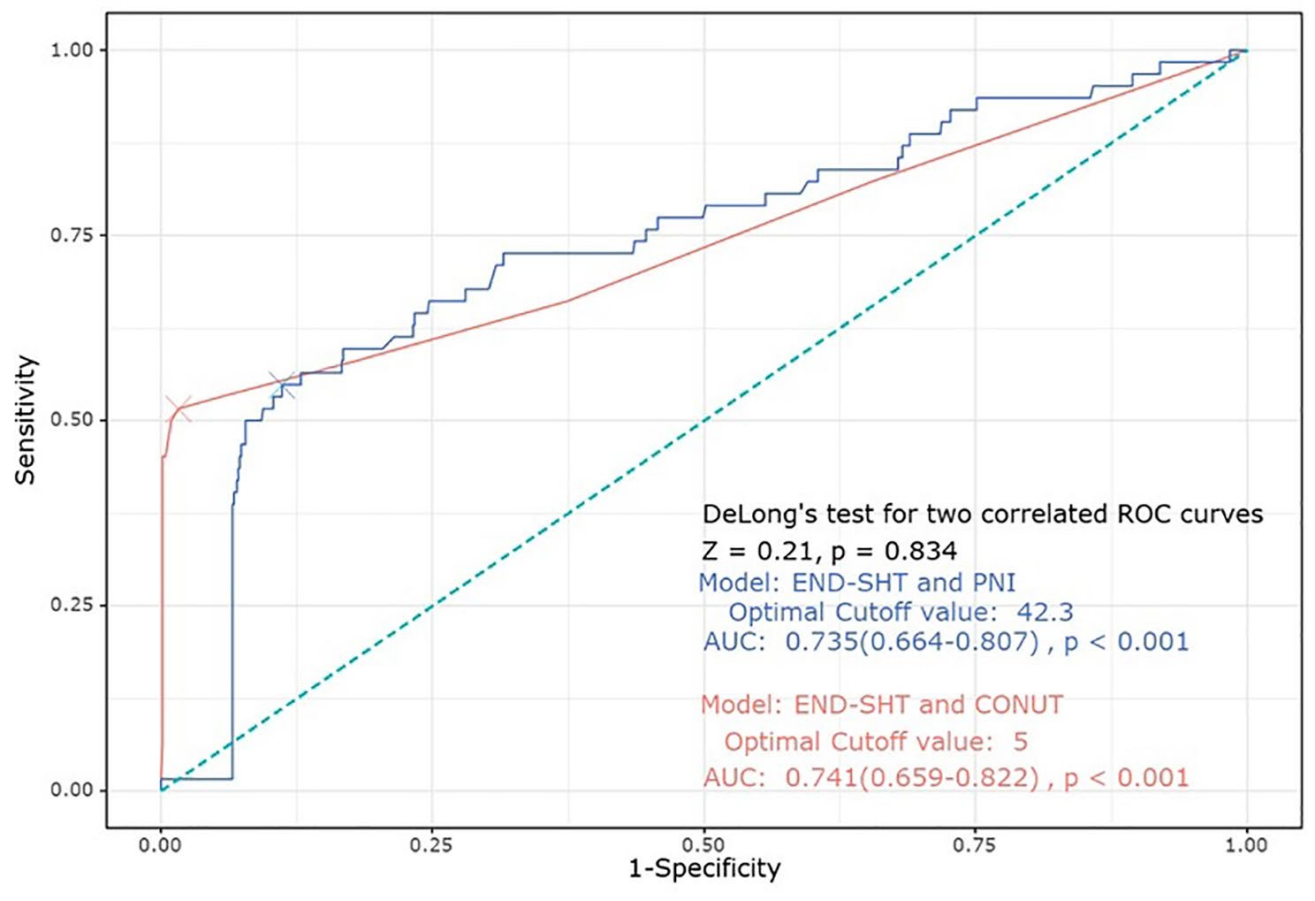
ROC curve showing the predictive ability of CONUT score and PNI for END-SHT Abbreviations: ROC, receiver operating characteristics; CONUT, controlling nutritional status; PNI, prognostic nutritional index; END-SHT, symptomatic hemorrhagic transformation

The ROC curve showed that the predictive ability of CONUT score and PNI for END-SHT was close to good (AUC of CONUT: 0.74, 95% CI [0.66–0.82]. p < 0.001; AUC of PNI: 0.74, 95% CI [0.66–0.81], p < 0.001). There were no significant differences in the prediction of END-SHT between CONUT and PNI levels. The cutoff values of the CONUT score and PNI were 5 and 42.3, respectively, for END-SHT (Fig. 3).

## Discussion

The main findings of this study are as follows: (1) Patients with worse malnutrition status, as estimated by both CONUT score and PNI, had a higher occurrence of END-SHT and worse stroke outcomes in acute ischemic stroke patients after IVT; (2) In multivariable analysis, malnutrition was associated with increasing the risk of END-SHT and poor stroke outcomes after IVT; and (3) the predictive ability of CONUT and PNI scores for END-SHT was reliable, and we suggest the cutoff values (CONUT score: 5; PNI: 42.3) of both nutritional markers for predicting END-SHT. CONUT score and PNI were initially reported to be effective predictors of nutritional status in malignancy, cardiovascular disease, and heart failure [19–23]. Recently, both markers have been suggested to be useful for predicting outcomes after acute ischemic stroke [24].

The frequency of malnutrition was 39.6% in this study, consistent with that of previous studies [13, 25, 26]. Although malnutrition is not a rare condition in acute ischemic stroke, clinicians may lack knowledge about the importance of nutritional status in stroke management [27]. The recent guidelines for acute ischemic stroke recommended that all stroke patients should be evaluated for individual baseline nutritional status, and malnutrition should be corrected as soon as possible [28]. The increased workload of evaluating nutritional markers in clinical practice could lead to this phenomenon. Hence, the easy estimation of nutritional markers using the CONUT score and the PNI could be suitable and feasible options in acute stroke settings. Several previous studies on this issue revealed that malnutrition, as estimated by the CONUT score and PNI, was associated with short- and long-term stroke prognosis,[7, 24, 29–31] but the prognosis after IVT remains unclear. Based on the results of a previous study that malnutrition estimated by PNI could increase poor stroke outcomes after IVT,[13] our main novel finding is that both CONUT score and PNI could be used to predict bleeding risk after IVT.

Inflammatory reactions and oxidative stress after ischemic stroke play a major role in stroke pathophysiology. Especially in IVT-treated stroke patients, these pathophysiologic reactions could increase bleeding tendency and infarct volume through blood-brain barrier breakdown and aggravating microangiopathy [32]. Serum albumin, as a multifunctional protein, plays neuroprotective roles in ischemic stroke by reducing erythrocyte aggregation and exhibiting an antioxidant effect [33]. In the inflammatory reaction, lymphocyte infiltration in the ischemic area could release pro-inflammatory cytokines and cytotoxic substances. Lower lymphocyte levels have been associated with poor functional outcomes after acute ischemic stroke [24, 34]. Although the association between total cholesterol and stroke outcomes remains unclear, low total cholesterol generally plays a role in promoting endothelial injury via arterial medial layer smooth muscle cell necrosis [35]. Therefore, we suggest that the detrimental pathophysiologic process in malnutrition could enhance the bleeding risk and poor stroke outcomes after IVT. Among the several reported nutritional markers, CONUT score and PNI reflecting nutrition and inflammation in subjects could be more reasonable nutritional markers for predicting HT after IVT.

Notably, the ROC curve showed that both CONUT score and PNI were useful for predicting HT after IVT and revealed the suggested cutoff values for both markers in our study. The optimal cut-off value of the CONUT score for predicting HT after IVT was 5. Previous studies showed that a CONUT score ≥ 5 (moderate to severe malnutrition state) could be associated with a poor 3-month functional outcome and mortality [24, 29]. Meanwhile, the optimal cutoff value of PNI for predicting HT after IVT was 42.3, which is reasonable compared to a previous study with an optimal cutoff value of 44.2 for 3-month outcomes after IVT [13]. Our novel findings showed that the different predictive values of CONUT score and PNI highlight the need for constantly updating the IVT strategy and guidelines.

The secondary outcomes were partly consistent with those of prior studies on CONUT score and PNI. Interestingly, malnutrition was associated with early stroke progression after IVT in the present study. The higher frequency of comorbidities, higher stroke severity, and unfavorable laboratory findings in the malnourished subjects in our study could explain this phenomenon. Notably, malnourished subjects had more LAA and CE and fewer SVO stroke subtypes, which was also found in a previous study [13]. Stroke progression contributing to END after IVT in LAA and CE stroke subtypes may be related to failed reperfusion, thrombus migration, re-occlusion, and recurrent embolism [36, 37]. Hence, the discrepancy of stroke subtypes according to the nutritional state could also affect stroke progression. Previous studies with general stroke populations and IVT-treated subjects using a single parameter, such as BMI, showed contradictory results between obesity and stroke outcomes (obesity paradox) [38, 39]. However, previous studies using nutritional parameters addressed this issue and revealed relatively consistent results, especially in IVT-treated subjects. We suggest that parameters reflecting multifactorial pathophysiologic processes could be more reliable compared to single parameters in assessing stroke outcomes in stroke patients. Nonetheless, further studies are warranted to explore the association between malnutrition and stroke outcomes.

Despite its consecutive multicenter registry-based nature, this study has several limitations. First, we retrospectively collected clinical data from the registry database despite the relatively large sample size. Second, the CONUT score and PNI were not evaluated at discharge or the delayed stroke stage. In addition, nutritional information such as dietary intake, weight change during hospitalization, and weight change after rehabilitation were not available in our study. However, since the main aim of this study was to evaluate acute stroke outcomes at hospitalization, serial changes in nutritional status were inevitable in our study. Third, the relatively small sample size of subjects with moderate-to-severe malnutrition could hinder the generalization of our results. However, our crude data suggest an epidemiological prevalence of severely malnourished subjects treated with IVT in future studies. Fourth, most of the variables were badly biased toward moderate-to-severe malnutrition. Although we adjusted these variables in the multivariable model, balancing the variables using propensity score matching may be needed in our analysis. However, with our worse clinical findings in malnourished subjects, clinicians may be more interested in establishing stroke interventions for those populations in clinical practice. Lastly, although we adjusted for several variables that may affect the outcomes, unmeasured confounding factors (e.g., muscle mass, nitrogen balance, and body composition) could hinder the applicability of our main findings.

## Conclusion

We suggest that nutritional status, as assessed by the CONUT score and PNI, could be associated with HT and poor stroke outcomes after IVT. The joint use of both markers, which are easily tested in acute stroke settings, could reasonably predict HT after IVT. Further prospective studies with larger sample sizes are needed to address the practical application of the CONUT score and PNI in clinical settings.

## Electronic supplementary material

Below is the link to the electronic supplementary material.

Supplementary Material 1

## Data Availability

The datasets used and/or analysed during the current study are available from the corresponding author on reasonable request.
