# Supplementary material for: The association between malnutrition status and hemorrhagic transformation in patients with acute ischemic stroke receiving intravenous thrombolysis
Source: BMC Neurol. 2023 Mar 14;23:106. doi: 10.1186/s12883-023-03152-3 (PMC10012700; doi:10.1186/s12883-023-03152-3)
Supplement: Supplementary file 1 — Supplementary Material 1 [file 12883_2023_3152_MOESM1_ESM.docx]

Supplementary table

Supplementary Table 1. CONUT scoring system

|  | Normal | Mild | Moderate | Severe |
| --- | --- | --- | --- | --- |
| CONUT. point | 0-1 | 2~4 | 5~8 | 9~12 |
| Serum albumin (g/dL) | ≥3.5 | 3.0-3.49 | 2.5-2.99 | <2.5 |
| Score | 0 | 2 | 4 | 6 |
| Lymphocyte count (x109/mm3) | ≥1.6 | 1.2-1.59 | 0.8-1.19 | <0.80 |
| Score | 0 | 1 | 2 | 3 |
| Total cholesterol (mg/dL) | ≥180 | 140-179 | 100-139 | <100 |
| Score | 0 | 1 | 2 | 3 |

Supplementary table 2. Multivariate analysis showing the impact of CONUT score on 3-month mortality.

|  | OR | 95% CI | p-value |
| --- | --- | --- | --- |
| CONUT scoring system |  |  |  |
| normal | ref |  |  |
| mild | 0.80 | 0.37-1.73 | 0.58 |
| moderate to severe | 2.87 | 1.03-7.99 | 0.04 |
| Age | 1.05 | 1.02-1.08 | 0.004 |
| Male | 0.56 | 0.27-1.14 | 0.11 |
| Initial NIHSS | 1.08 | 1.02-1.14 | 0.004 |
| Stroke mechanism | 1.62 | 1.09-2.42 | 0.02 |
| Previous stroke | 1.36 | 0.59-3.11 | 0.47 |
| DM | 0.60 | 0.29-1.25 | 0.18 |
| CAD | 2.29 | 0.90-5.86 | 0.08 |
| Atrial fibrillation | 1.43 | 0.70-2.91 | 0.33 |
| Previous use of antithrombotics | 0.83 | 0.39-1.78 | 0.63 |
| tPA dose, | 3.34 | 0.68-16.38 | 0.14 |
| Total cholesterol | 0.996 | 0.98-1.01 | 0.60 |
| Albumin | 1.04 | 0.52-2.08 | 0.91 |
| Hemoglobin | 1.06 | 0.87-1.29 | 0.56 |
| Platelet | 1.001 | 0.997-1.01 | 0.57 |
| Creatinine | 1.49 | 0.99-2.26 | 0.06 |
| LDL | 1.01 | 0.99-1.03 | 0.29 |
| PT | 1.14 | 0.12-10.85 | 0.91 |
| CRP | 1.02 | 1.002-1.03 | 0.03 |

Supplementary table 3. Multivariate analysis showing the impact of PNI on 3-month mortality.

|  | OR | 95% CI | p-value |
| --- | --- | --- | --- |
| Low PNI | 1.10 | 1.34-3.57 | 0.046 |
| Age | 1.05 | 1.01-1.08 | 0.01 |
| Male | 0.60 | 0.31-1.18 | 0.14 |
| Initial NIHSS | 1.07 | 1.02-1.12 | 0.01 |
| Stroke mechanism | 1.61 | 1.09-2.38 | 0.02 |
| Interval from arrival to IVT | 1.004 | 0.997-1.014 | 0.24 |
| Atrial fibrillation | 1.34 | 0.68-2.61 | 0.4 |
| tPA dose | 2.49 | 0.55-11.20 | 0.24 |
| Albumin | 0.66 | 0.34-1.31 | 0.24 |
| Creatinine | 1.45 | 0.97-2.15 | 0.07 |
| LDL | 1.00 | 0.99-1.01 | 0.45 |
| CRP | 1.02 | 1.00-1.03 | 0.03 |
| Initial random glucose | 1.00 | 0.996-1.01 | 0.85 |
